# Fabricating PCPDTBT/PC61BM organic solar cells using the PVD method

**DOI:** 10.1016/j.heliyon.2024.e41561

**Published:** 2025-01-10

**Authors:** Or Gindi, Zeev Fradkin, Anat Itzhak, Sofiya Kolusheva, Peter Beker

**Affiliations:** a Department of Electronic Engineering, Shamoon College of Engineering, Ashdod, 77245, Israel; b Department of Chemical Engineering, Shamoon College of Engineering, Ashdod, Israel; c Department of Chemistry, Bar-Ilan University, Ramat Gan, 5290002, Israel; d Ilse Katz Institute for Nanoscale Science and Technology, Ben Gurion University, Israel; e Copernicus Institute of Sustainable Development, Utrecht University, the Netherlands

**Keywords:** OPV, PVD, XPS, ESEM, PCPDTBT, PC61BM, Solar cells, IV, FTIR, XRD, Renewable energy

## Abstract

A study conducted to investigate the fabrication of organic solar cells based on PC61BM:PCPDTBT using the Physical Vapor Deposition (PVD) method. The research carries out a number of analyses for structural evaluations (XRD and ESEM), compositional change (XPS and FTIR), and functional assessment (absorption and I-V testing), with the goal of understanding the materials’ properties, their behavior during deposition and the resulting characteristics of the fabricated solar cells. While demonstrating promise, the PVD method faces challenges in the maintenance of electrical attributes during vaporization, and the need for further optimization. The findings highlight both the advantages and limitations of the method and point the way toward achieving optimal outcomes in the manufacture of organic solar cells using PVD. The main scientific contribution of this manuscript lies in the application of the PVD method for fabricating PCPDTBT. The final device features a working solar cell device made of the materials discussed above with Cell size of $0.0026\;mm^{2}$, cell efficiency of about 0.18% and thickness of 309 nm–400nm. These results showcasing the possibility of building and fabricating organic cells with the PVD method.

## 1 Introduction

The pressing need for clean and renewable energy solutions has led to the emergence of new technologies, one of them in the solar cell development is organic photovoltaics (OPV) marked as a promising alternative to conventional silicon solar cells. By utilizing organic semiconducting polymers and fullerenes, OPVs have the potential to offer a more affordable and less environmentally taxing photovoltaic technology. Despite the challenges in achieving sufficient device stability and efficiency, OPVs' significant benefits and possibilities have propelled their research and development. Photovoltaic solar energy is today the dominant renewable technology, and silicon solar cells account for more than 90 % of the PV industry [1]. However, silicon solar cell fabrication is complicated, relatively expensive, and requires strict conditions, including high temperatures and precious metals. Furthermore, other widely used PV materials, such as cadmium telluride (CdTe) and copper indium gallium selenide (CIGS), have raised environmental concerns due to their toxic nature [2]. OPVs have in recent decades emerged as a promising alternative in view of their affordability and minimal material usage, as well as their potential for lower production costs and a broader scope of application. Unlike traditional solar cells, OPVs utilize organic semiconductors, typically blends of semiconducting polymers and fullerenes, which exhibit similar physical properties to inorganic semiconductors. The unique attribute of OPVs is their ability to be fabricated on various lightweight, flexible substrates, significantly expanding their application possibilities, which range from portable devices to office building windows and electric car vehicles to household rooftops. Moreover, OPVs can be produced using less energy-consuming fabrication methods, contributing to their environmental and economic advantages. The current state of the art organic solar devices [3,4] using PCPDTBT:PCBM can reach efficiencies between 4% and 6% and FF around 55 %. These efficiencies are mainly achieved in a Bulk Heterojunction (BHJ) cellconfiguration which utilize a blend of the donor and acceptor materials in a mixed solution, that offers improved exciton dissociation efficiency by maximizing the donor-acceptor interface area. The poor performance compared to standard BHJ devices may be attributed to the smaller interfacial area for exciton dissociation in the bilayer configuration used here and potential challenges associated with the PVD deposition. Wangyao Ge in Ref. [5] and Adeoye in Ref. [6] showcased the benefits of this method and made a similar research test conditions of PCPTCBT:PC71BM device using laser deposition. Showing similar results with material evaporation deposition and progress with this field. The PVD research showcased here is using different materials and a more energy efficient approach for this technique while using the bottom-up fabrication method. The most common way of making these devices today is by chemical solutions processes and spin coating and Research also shows the need for an extra hole-transport layer or the use of BHJ for better conductivity and carrier extraction. The state-of-the-art for organic photovoltaics showing good results reaching up to 18 % efficiency in lab conditions. However, these methods has limitations, such as quality control as the area for spin coating is increased and the lack of precision in the case of printing. Moreover, concerns have been raised with respect to certain materials required in these processes. While PVD is less commonly used due to the large size of the organic molecules involved and the associated high temperatures required for evaporation, it offers its own advantages [7] such as layer by layer (LBL) deposition. These include the ability to produce a thin, organized structure of materials without mixing or generating waste products, efficient material usage, and precise control over layer thickness and ratio [8,9]. PVD method is a bottom-up self-assembly process in which smaller system components assemble themselves to form larger, more complex structures. The result is the creation of rigid nanoscale materials and structures in which systems and structures are built up from the molecular or atomic level. As the vaporized source material lands on the target surface, it condenses into a solid form and forms a thin layer. The particles of the source material may have a specific orientation or arrangement that causes them to self-assemble into complex structures as they land on the target. The specific nature of the self-assembly will depend on the properties of the source material and the conditions under which the process is carried out [10,11].

The experiments presented in this study uses the PVD method to create an OPV consisting of PC61BM (a fullerene) and PCPDTBT (a polymer) for the organic P-N junction. While the use of PVD for OPV fabrication is novel, its specific impact lies in addressing challenges such as scalability, cost-effectiveness, and material stability. Unlike traditional solution-based methods, PVD offers a unique approach that allows precise thickness control and eliminates solvent waste, which could improve long-term device stability. This study represents a significant step forward by demonstrating that, with further optimization, PVD can contribute to large-scale OPV production with enhanced film quality.

## 2 Experimental setup

A series of tests was conducted in order to monitor the characteristics of the materials during the process and to evaluate the functionality of the finished product as an operating solar cell. The initial tests included: Thermogravimetric analysis (TGA), X-ray diffraction analysis (XRD), X-ray photoelectron spectroscopy (XPS), and environmental scanning electron microscope (ESEM), with the goal of determining the conditions of cell manufacturing and whether an even crystalline layers of the materials has been achieved. Optical absorption and I-V measurements were then performed in order to evaluate the functioning of the cell.

### 2.1 Physical vapor deposition

A custom-built vacuum chamber was used for the PDV process. It contains a top clamp to hold the glass samples on which the materials were deposited, and a tub where the material was placed. The tub was connected to a heater that can reach temperatures of up to 500C. PVD provides a balance of high purity, precise control over film thickness, and reasonable scalability. For instance, PVD's ability to deposit clean, defect-free films without solvent contamination is advantageous for ensuring consistent performance, particularly in organic photovoltaics where interface quality is crucial for efficient charge separation and transport. The flexibility of PVD makes it ideal for developing LbL deposition strategies, which can improve the phase separation between donor and acceptor materials, thereby enhancing device efficiency in comparison to other thin-film deposition techniques like solution processing, Chemical Vapor Deposition (CVD), and Atomic Layer Deposition (ALD), it offers distinct advantages in terms of purity, precision, and the quality of deposited layers. For example: Solution-based methods, such as spin coating, are popular for their cost-effectiveness and scalability. However, solution processing often faces challenges related to phase separation and the lack of precise morphology control. Also, CVD often involves reactive gases that can lead to undesirable by-products and affect the quality of the deposited film. The high temperatures required for CVD can also degrade sensitive organic materials like PCPDTBT. The review by Xue et al. (2018) indicates that while Atomic layer deposition (ALD) can be effective for creating buffer layers with very high precision, PVD strikes a practical balance by enabling precise, high-quality layer formation without the significant cost and time associated with ALD [12,13].

### 2.2 Thermogravimetric analysis (TGA)

TGA measurements were performed using a TA Instruments Q500 Thermogravimetric Analyzer (TGA) in an N2 atmosphere. It is able to measure weight changes in a material as a function of temperature (or time) under a controlled atmosphere. One of its main uses is to measure a material's thermal stability and composition. Samples were held in an alumina crucible and heated up to 1000C with a heating rate of 10C/min.

### 2.3 X-ray diffraction analysis (XRD)

X-ray diffraction (XRD) is a technique used for the structural analysis of crystalline materials. The principal mechanism of the test is the scattering of incident X-rays by a material's crystal lattice. An XRD pattern or diffractogram reveals the resulting diffracted X-ray intensities versus diffraction angle (2θ). This pattern is unique to the crystal structure and atomic arrangement of the sample material and can be analyzed to determine various properties of the material. In this test, we used the Bruker D8 Discover diffractometer which includes the linear energy discriminate detector LINXEYE XE.

### 2.4 X-ray photoelectron spectroscopy (XPS)

XPS is a surface-sensitive technique that provides information about the elemental composition, chemical state, and electronic state of the materials within the top ∼1–10 nm of the sample's surface. An XPS measurement results in a spectrum of the number of photoelectrons (i.e. their count) versus their kinetic energy. This spectrum can be analyzed to provide quantitative elemental composition and identify the chemical state of the elements. We used the X-ray photoelectron spectroscopy-Auger electron spectroscopy (XPS/AES) ESCALAB 250 ThermoFisher Scientific, a multi-technique instrument that can carry out XPS and AES surface-sensitive analysis. This system optimizes the routine high-speed XPS analysis of large areas [14].

### 2.5 Environmental scanning electron microscope (ESEM)

ESEM is a technique to image samples in their natural or “wet” state without requiring extensive sample preparation, such as coating the sample with conductive material. The Quanta 200 FEG E ESEM was used in the experiment. It employs a field-emission gun (FEG) electron source in an exceptionally high chamber-pressure environment [15].

### 2.6 FTIR

The FTIR spectra reported here were all recorded using a Nicolet 8700 FTIR spectrometer fitted with a DTGS detector with ATR diamond holder. All single-beam spectra were measured against a background recorded from clean crystal. The spectra were recorded in the range of 4000 to 700 cm-1, at 4– resolution and 64 scans with optical velocity – 0.4 and aperture – 100. The FTIR data were collected using the OMNIC software. All results were plotted after atmospheric correction. FTIR measurement of materials is a powerful technique for molecular characterization and detection. The spectra are related to the molecule's vibronic structure and provide the compound's fingerprinted signature.

### 2.7 Optical absorption

Absorption experiments were conducted using a Cary 5000 high-performance UV–Vis–NIR spectrophotometer in the wavelength range of 175–3300 nm, which includes a Pbsmart detector.

### 2.8 I-V test

I-V testing is the most widely used technique for analyzing solar cell characteristics, such as current-voltage operating point and device power output. We used it to determine whether the final product behaves like a solar cell and can produce current under illuminating light. A laser connected to an optic fiber was used to illuminate a small portion of the cell and a Keithley 2400 SMU to manipulate the voltage through the cell. We measured the corresponding current and analyzed it using software built with LabVIEW.

## 3 Results and discussion

### 3.1 XRD

**Fig. 1:**
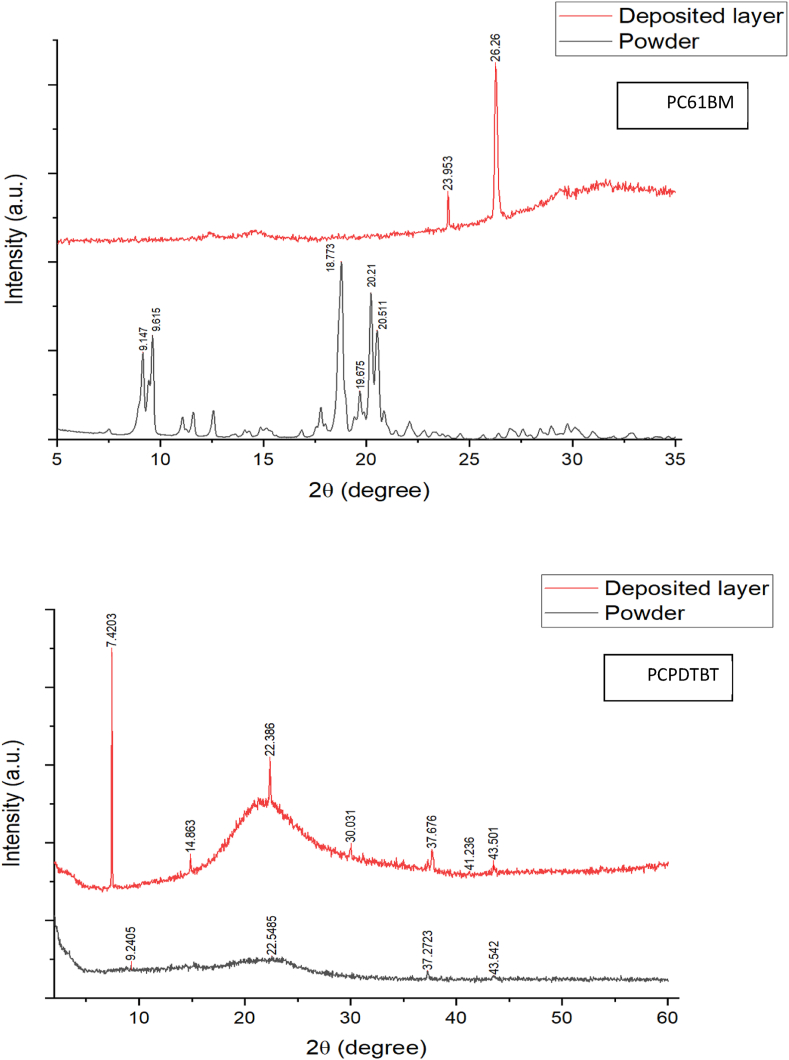
XRD results for PC61BM (top) and PCPDTBT (bottom).

X-ray diffraction (XRD) analysis was conducted on the PCPDTBT and PC61BM materials in powder form and after deposition, Fig. 1. XRD operates by scattering X-rays off a crystal lattice, utilizing the principles of constructive interference and Bragg's Law. When X-rays encounter crystalline material, they interact with the atoms in the lattice. If the scattered X-rays are in phase, a distinct diffraction pattern is produced. The specific conditions for this constructive interference depend on the arrangement of the crystal and the wavelength of the X-rays used. In contrast, amorphous materials, which lack long-range atomic order, produce broad and diffuse XRD patterns instead of the sharp peaks characteristic of crystalline structures. This difference arises because the irregular atomic arrangement inhibits constructive interference at defined angles.

XRD is essential for assessing material structure, especially following processes such as physical vapor deposition (PVD), as it helps confirm whether the material is crystalline or amorphous. Additionally, Energy Dispersive X-ray Spectroscopy (EDX) aids in identifying various phases of a material before and after deposition by linking elemental data to specific crystalline structures. While EDX alone does not directly provide insights into crystallite size, its combination with XRD can reveal clues about size through the Scherrer effect, which relates peak broadening to crystallite dimensions. Moreover, the integration of XRD and EDX can enhance our understanding of how strain influences elemental distribution, indirectly aiding in the analysis of lattice strain [[16], [17], [18]]. Upon comparing the results with those in Refs. [19,20], it was evident that the XRD patterns from the powders exhibited a match for both materials. However, in the samples deposited, distinct peaks and a plateau were observed, indicating the occurrence of crystallization within the materials. This intriguingly suggests the formation of a new state of mind. In the case of PC61BM (top), two prominent peaks were observed at 23.95 and 26.26 (2θ). Similarly, in the case of PCPDTBT (bottom), two major peaks were evident at 7.42 and 22.38 (2θ), which represent the crystallized layers. These peaks were consistent with the PCPDTBT XRD pattern reported in the references. The presence of a hump shape at the bottom was due to the glass substrate on which the materials were deposited, and this artifact can be disregarded. It is noteworthy that both materials were applied onto an amorphous glass substrate. The high prominence of the peaks indicates successful crystallization, implying that a crystallized layer of materials had formed over the glass substrate. This significant finding demonstrates the capability of the Physical Vapor Deposition (PVD) method to arrange materials into an organized layer, ultimately altering X-ray diffraction (XRD) is a technique used for the structural analysis of crystalline materials. The main mechanism of the test is the scattering of incident X-rays by a material's crystal lattice. An XRD pattern or diffractogram reveals the resulting diffracted X-ray intensities versus diffraction angle (2θ). A monochromatic X-ray source typically provides a consistent wavelength, improving peak sharpness and intensity. The step size determines the resolution of the diffraction pattern; smaller step sizes yield finer resolution but may increase measurement time. Together, these factors affect the ability to discern subtle features in the diffraction pattern. This pattern is unique to the crystal structure and atomic arrangement of the sample material and can be analyzed to determine various properties of the material. In this test, we used the Bruker D8 Discover diffractometer which includes the linear energy discriminate detector LINXEYE XE. This detector enhances resolution and sensitivity through its advanced technology. It offers improved energy resolution of less than 380 eV, allowing for better differentiation between X-ray energies, which reduces background noise. Additionally, its fast data acquisition rate increases analytical efficiency, enabling the detection of weak signals that might otherwise be missed by traditional detectors.

### 3.2 TGA

**Fig. 2:**
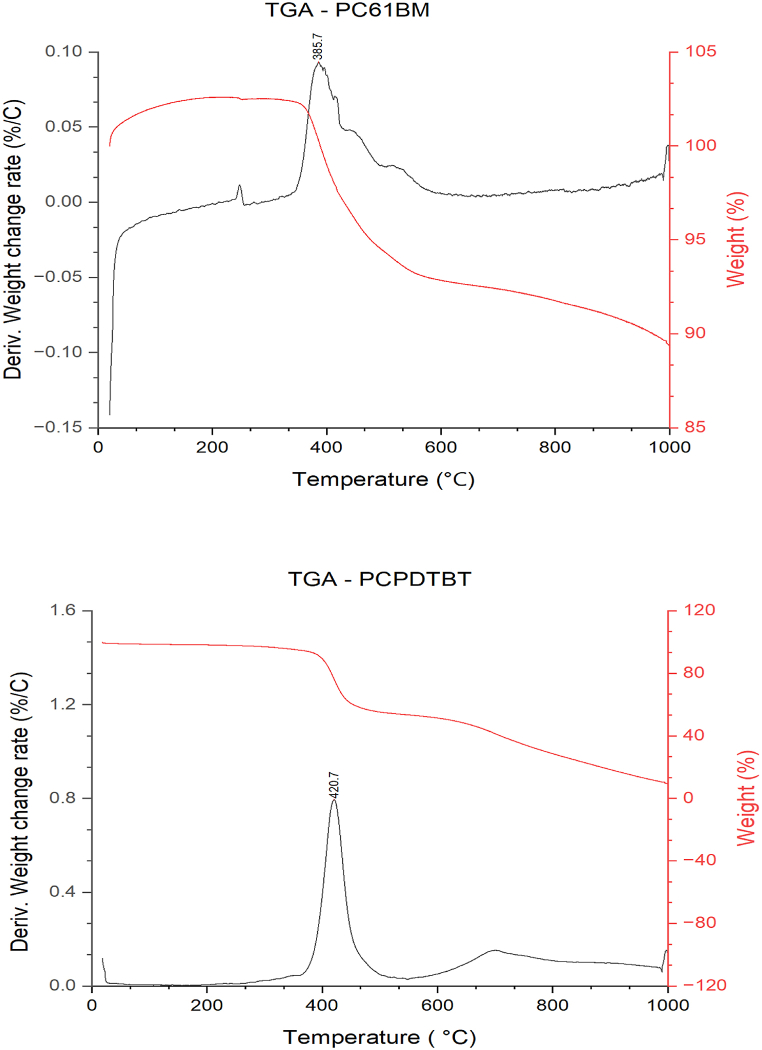
TGA analysis for PC61BM (top) and PCPDTBT (bottom).

This test was made to identify the temperatures at which PC61BM and PCPDTBT display the highest evaporation rates. The results are presented in Fig. 2 and clearly show that PC61BM exhibited its most rapid evaporation at approximately 385C. At this specific temperature, the material's weight loss rate reached a maximum, suggesting a significant release of volatile components. Similarly, the peak evaporation rate for PCPDTBT was observed at around 420C. This temperature point marked the stage in which the material experienced the most substantial mass loss due to evaporation, indicating the liberation of volatile compounds or decomposition products. Identifying these critical temperature points is of great importance in various applications, such as material processing, formulation design, and understanding the thermal stability of organic materials. Knowing the temperatures at which these materials evaporate most efficiently allows researchers to optimize processing conditions and enhance the performance of devices or materials that incorporate PC61BM or PCPDTBT.

### 3.3 ESEM

**Fig. 3:**
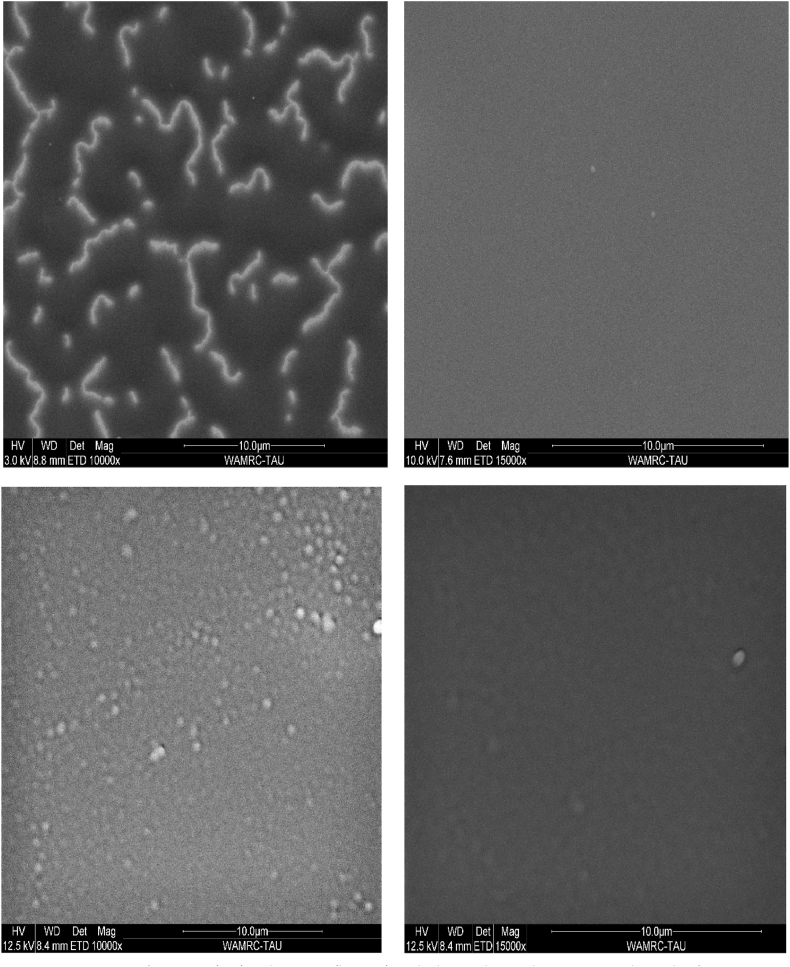
SEM image of PCPDTBT (top) and PC61BM (bottom) applied on a silicon substrate. For each couple of images PCPDTBT and PC61BM we can see an improvement in the layer uniformity from left to right for different depositions. After reaching the uniform layers with PVD by implementing the results from TGA analysis we could get a cell with less defects.

Environmental Scanning Electron Microscopy (ESEM) was used to assess the uniformity and surface roughness of the deposited layers. A more uniform layer indicates fewer defects. The two top images of Fig. 3 show the ESEM analysis of PCPDTBT and reveal that the initial deposition did not fully cover the surface, resulting in a fiber-like structure. The material did not settle in some areas, leaving noticeable bald spots. However, increasing the substrate temperature achieved a more uniform layer. The bottom two photos depict the PC61BM layer [21] and show that our deposition technique led to nanosized cluster domains with minimal micron-sized clusters across the film surface [22]. This observation highlights the successful outcome of the deposition process in generating the desired nanoscale features with limited large clusters [23].

### 3.4 XPS

The X-ray photoelectron spectroscopy (XPS) test results for PCPDTBT and PC61BM reveal a significant phase transition in the materials after vapor deposition. For PC61BM, the Si2p peak shifted from 104.65 eV to 104.15 eV, the C1s peak shifted from 284.81 eV to 284.98 eV, and the O1s peak shifted from 533.00 eV to 532.58 eV, indicating notable chemical changes. In the case of PCPDTBT, the S2p peak shifted from 167.92 eV to 167.18 eV, the C1s peak shifted from 285.58 eV to 285.12 eV, the N1s peak shifted from 400.02 eV to 399.74 eV, and the O1s peak shifted from 532.60 eV to 533.17 eV. Interestingly, an Si2p peak at 105.87 eV appeared after the deposition, but this can be attributed to the silicon from the substrate surface rather than the organic material itself. These shifts suggest parallels to classic structural phase transitions typically observed in organic and inorganic crystals, which are defined primarily by minute atomic displacements. Such phase transitions in organic compounds often result from variations at the molecular level, leading to significant changes in molecular crystal symmetry. Depending on the nature of the molecular transformations, they can be irreversible, as in the case of covalent bond changes, or reversible in the case of weaker interactions. In the context of PCPDTBT and PC61BM, the observed XPS shifts suggest a conformational transition influenced by molecular interactions reminiscent of changes observed in peptide nanotubes when subjected to physical vapor deposition. Such transitions can alter organic solar cells' electronic properties and device performance [24]. Oxygen presence was observed in the PCPDTBT peaks even though there is no oxygen in the chemical structure of the molecule. This can be explained by oxidation of the sample between the time of deposition and the XPS.

#### 3.4.1 Elemental ID and quantification

PC61BM powder:

**Table:** 

Name	Peak BE	FWHM eV	Area (P) CPS.eV	Atomic %	Q
Si2p	104.65	0.04	61.33	0.33	1
C1s	284.81	0.88	21011.84	94.66	1
O1s	533.00	2.83	3049.27	5.01	1

PC61BM after deposition:

**Table:** 

Name	Peak BE	FWHM eV	Area (P) CPS.eV	Atomic %	Q
Si2p	104.15	3.00	545.66	2.19	1
C1s	284.98	1.80	24051.95	81.90	1
O1s	532.58	2.30	12800.71	15.91	1

PCPDTBT polymer powder:

**Table:** 

Name	Peak BE	FWHM eV	Area (P) CPS.eV	Atomic %	Q
S2p	167.92	3.26	6228.62	8.25	1
C1s	285.58	4.28	38420.18	85.78	1
N1s	400.02	2.76	3493.07	4.38	1
O1s	532.60	1.44	2022.06	1.59	1

PCPDTBT polymer after deposition:

**Table:** 

Name	Peak BE	FWHM eV	Area (P) CPS.eV	Atomic %	Q
O1s	533.17	2.66	27154.78	21.33	1
N1s	399.74	1.47	2874.59	3.61	1
C1s	285.12	2.22	27023.23	60.37	1
S2p	167.18	5.69	3508.77	4.65	1
Si2p	105.87	5.26	3717.40	10.04	1

### 3.5 FTIR

#### 3.5.1 PC61BM

**Fig. 4:**
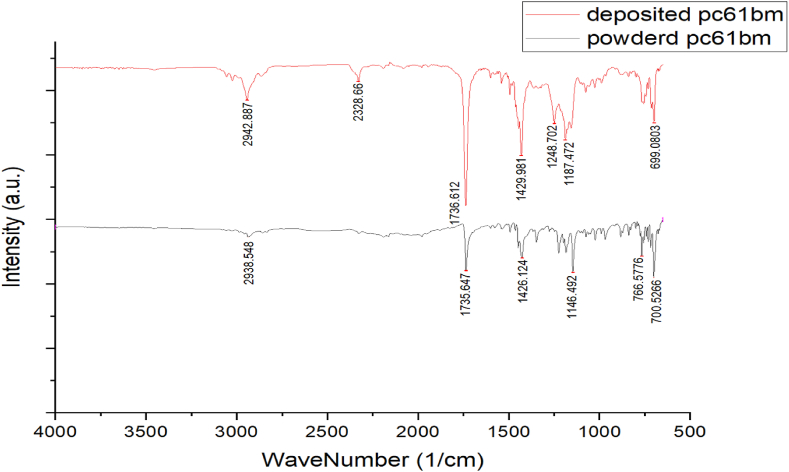
FTIR measurements for powdered PC61BM (bottom) and post-PVD (top).

The FTIR spectrum of PCB61M provides a detailed view of its molecular structure and the types of bonds present. Fig. 4 both datasets highlight the presence of the ester functional group with peaks around 1730–1740 cm⁻^1^ for C=O stretching and around 1240–1260 cm⁻^1^ for C-O trying. These peaks confirm PC61BM solubility enhancements in organic solvents. Additionally, the influence of alkyl chains, which play a role in PC61BM solubility and phase behavior, is evident from the C-H stretching peaks around 2900–2950 cm⁻^1^ and the C-H bending around 1400–1450 cm⁻^1^. The results for the powdered PC61BM show an unusual peak at 2328 cm⁻^1^, potentially as a result of external interferences like atmospheric CO₂, and specific fullerene-related vibrations at 1187 cm⁻^1^ and 699 cm⁻^1^. In contrast, the post-PDV data shows peaks associated with the phenyl group's vibrations, contributing to PC61BM's overall structure and solubility. Essentially, the FTIR spectrum of the PC61BM confirms its molecular structure [25].

#### 3.5.2 PCPDTBT

**Fig. 5:**
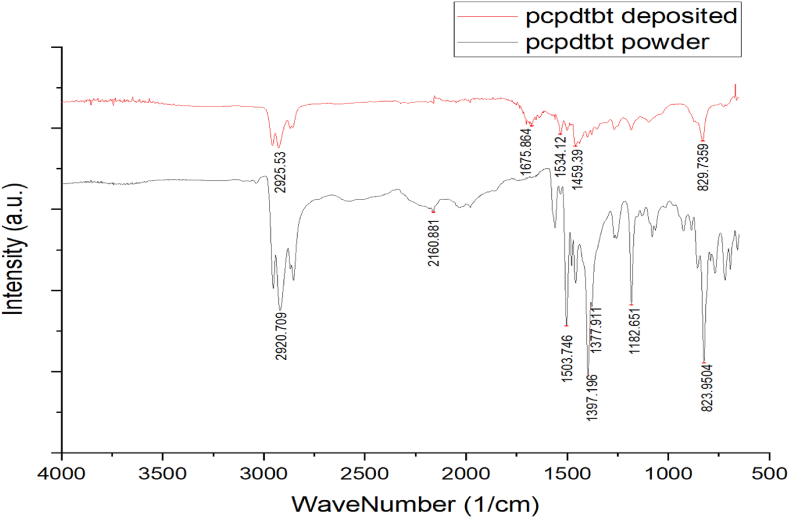
FTIR measurements for powdered PCPDTBT (bottom) and post-PVD (top).

As above, the FTIR spectrum provides a detailed view of PCPDTBT's molecular structure, and the types of bonds presented in Fig. 5. Typically, PCPDTBT exhibits C-H stretching vibrations due to alkyl chains around 2900–2950 cm⁻^1^, which are observed at 2925 cm⁻^1^. This confirms the presence of these chains, which enhance solubility. The peak at 1675 cm⁻^1^, which is consistent with the typical 1650–1700 cm⁻^1^ range, may indicate carbonyl functionalities or other double-bond vibrations. A peak around 1500–1600 cm⁻^1^ is characteristic of aromatic C=C stretching. The observation at 1534 cm⁻^1^ is consistent with this, indicating the polymer's conjugated backbone. The peak at 1459 cm⁻^1^, consistent with the typical C-H bending in alkyl groups around 1400–1460 cm⁻^1^, further confirms the alkyl side chains. Lastly, the peak at 829 cm⁻^1^ matches the typical out-of-plane C-H bending in aromatic rings around 800–850 cm⁻^1^. Overall, the FTIR results for PCPDTBT closely align with the expected peaks [26,27].

### 3.6 Absorption

**Fig. 6:**
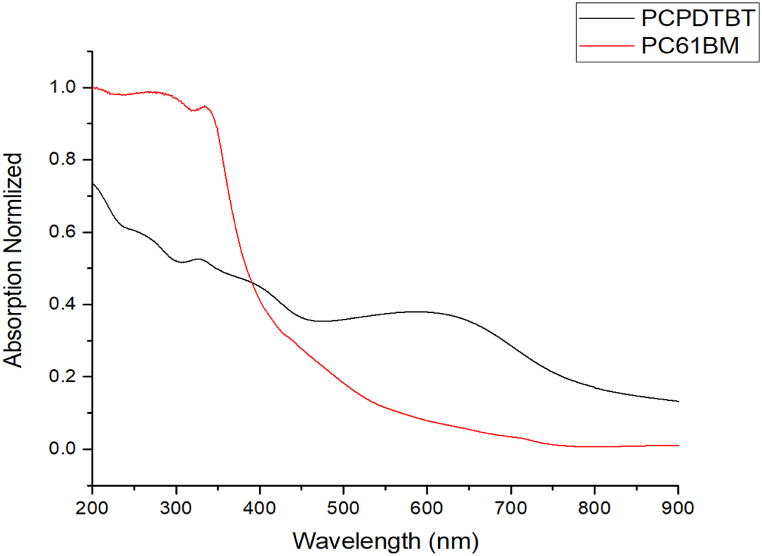
Absorption of PC61BM and PCPDTBT after deposition, by wavelength.

During the experiment, individual layers of PCPDTBT and PC61BM were meticulously deposited through vaporization within a vacuum chamber, thus ensuring each layer's purity and uniformity. The procedure was carried out separately for each material in order to maintain the integrity of both substances and their distinctive properties. Following the creation of the layers, a comprehensive analysis was undertaken to evaluate their absorption capabilities, a critical factor in determining efficiency in solar cells. The results are presented in Fig. 6. For the PCPDTBT layer, represented by the black line, two significant absorption peaks were observed: the first in the range of 200–500 nm and the second in the range of 500–700 nm. This dual-peak phenomenon indicates a broad spectrum of light absorption, a characteristic that can be leveraged to enhance the efficiency of solar cells. The PC61BM layer, represented by the red line, shows a pronounced absorption peak in a narrower range, i.e. 200-450 nm. This peak is indicative of the material's ability to absorb high-energy photons, a property that is essential in the conversion of sunlight to electricity. In the subsequent pivotal phase of the experiment, the two materials were integrated on a single substrate. This establishes a PN junction, which is a critical component of solar cells. This creates a space where the disparate band gaps of the two materials converge, facilitating the efficient flow of electrons and the creation of holes, a process central to electricity generation.

### 3.7 I-V testing

**Fig. 7:**
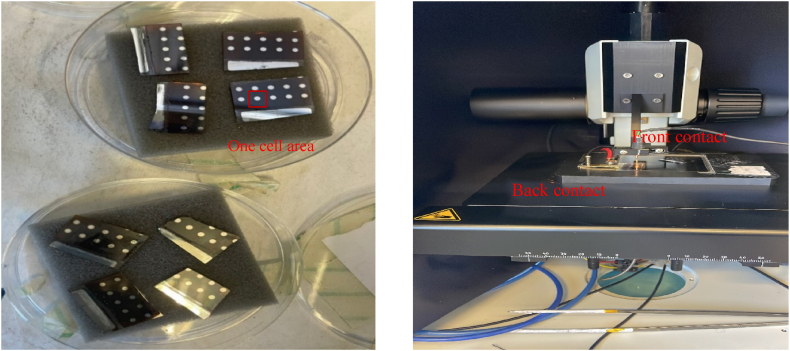
Left – Organic cells ready for I-V testing (cell size of 0.027 cm^2). Right – Solar Simulator used for testing based on a fiber-optic light source.

**Fig. 8:**
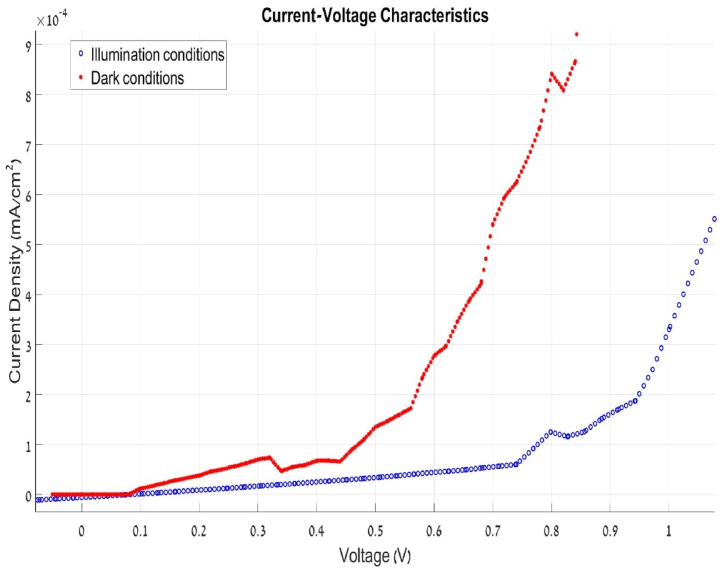
I-V testing of the device. Red line – dark conditions. Blue line – under illumination the dark test current density (blue line) is −7 × 10^ (−10) j/(cm^2) and the illumination current density (red line) is −2 × 10^(-6) j/(cm^2).

I-V measurements were performed on the completed cells. Fig. 7 shows the cells ready for testing and how the measurements were made inside the solar simulator. Fig. 8 shows the result of I-V measurement of the organic solar cell with current induced by the light source flowing through the cell. At $V_{oc}=0.6V$, The dark current density is $-7∙10^{-10}\frac{j}{cm^{2}}$ and the illumination current density is $-2∙10^{-6}\frac{j}{cm^{2}}$. The linear shape at the lower voltages suggests that the cell has a low shunt resistance value [28], implying that some current is lost inside the material instead of getting to the collectors. This might be due to the cell thickness of 309 nm–400nm which is on the thicker size of the usual 80 nm-150nmper layer causing low carrier mobility inside the device, which can be improved by adding materials that enhance the carrier's mobility and by controlling the layer thickness. Nevertheless, the results are more than sufficient to demonstrate the potential of using the PVD method to fabricate PCPDTBT/PC61BM solar cell devices.

### 3.8 Power output

**Fig. 9:**
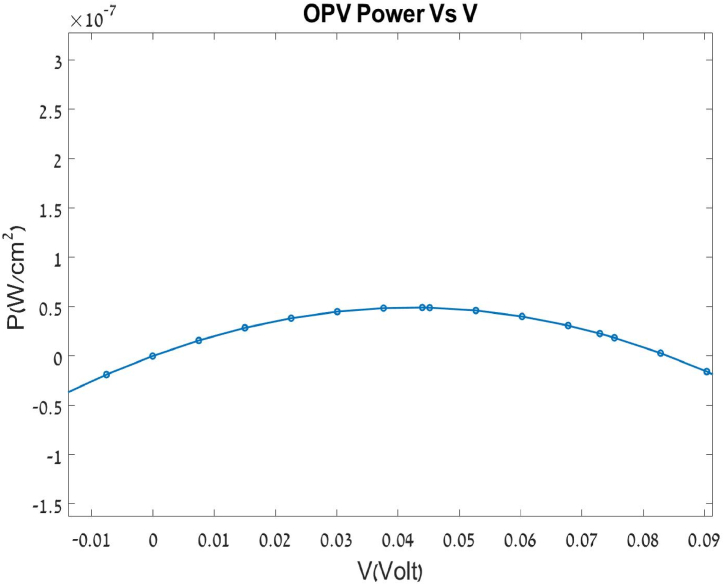
Cell Power output under illumination.

Fig. 9 presents the cell's power curve under illumination. Cell size is $0.0026\;mm^{2}$ and cell efficiency is about 0.18%, which was calculated based on an optic fiber light source with illumination of around 1 sun (AM 1.5). Though the efficiency is not particularly high, the results demonstrate the possibility of manufacturing an organic cell with PCPDTBT and PC61BM using the PVD method rather than the conventional chemical solution. The reason for the low power is primarily the low current, which may be associated with the high resistance seen in the I-V curve, which in turn is associated with layer thickness. Another reason may be the small surface contact between the materials which is where the charge carriers are created in an organic cell. Another approach is developing hybrid systems, where solution processing can be used for active layer deposition, complemented by PVD for electrode or interfacial layers, combining cost-effectiveness with precision. Kiran et [29] noted that layer-by-layer (LbL) deposition could also help in improving the vertical phase separation of donor and acceptor materials, which is crucial for efficient charge transport. By adopting these approaches, PVD could become a viable method for the commercial-scale production of OPVs, ensuring both high-quality films and scalability.” A significant limitation of PVD in the context of OPVs is its scalability, particularly in adapting laboratory-scale methods for industrial-scale applications. However, integrating roll-to-roll (R2R) processing with PVD could address these scalability challenges effectively, as highlighted by Gupta et [12] and Xue et [13]. This would allow for continuous and consistent deposition over large, flexible substrates, making PVD a more practical option for industrial production.

## 4 Conclusions

In conclusion, this study has demonstrated the feasibility of using PVD for fabricating OPVs using PCPDTBT: PC61BM, but the efficiency gap compared to other state-of-the-art methods remains. Analytical methods such as TGA and ESEM were used to determine optimal vaporization temperatures, which were found to be 420C for PCPDTBT and 390C for PC61BM. Subsequently, we carried out a series of analyses such as XRD, XPS, and FTIR to confirm the conservation of the materials’ intrinsic properties. The XRD analysis revealed distinct peaks post-deposition, indicating the formation of a crystalline layer on the substrate, while the FTIR analysis confirmed the retention of polymeric characteristics post-vaporization. In contrast, the XPS diagnostics showed altered chemical compositions, hinting at a phase transition in the structural framework of the substances. The technique is hampered by the potential loss of electrical attributes during the vaporization phase, which inhibits the envisioned formation of the PN junction resulting in cell efficiency of 0.18 % which is very low for a solar device. This could be a result of the cell thickness 300–400 nm. Similar methods research showed, like Wangyao Ge, showed efficiencies of 0.4–0.8 %. The absorptive capacity exhibited a mild reduction in the 500–700 nm region of the PCPDTBT spectrum, although a peak in that region demonstrated continued absorption. Future work should focus on optimizing deposition conditions, such as introducing low-energy deposition techniques or using co-deposition with additives to improve layer intermixing. Addressing these factors could bridge the gap between the efficiencies of PVD-fabricated devices and those using high-efficiency non-fullerene acceptors, making PVD a competitive approach for the next generation of organic solar cells.

This is early proof-of-concept. Future work will focus on improving efficiency through optimizing layer thickness, or co-deposition techniques to enhance the morphology and charge separation capabilities of the device.

## CRediT authorship contribution statement

**Or Gindi:** Writing – original draft, Investigation, Formal analysis. **Zeev Fradkin:** Validation, Supervision, Project administration, Conceptualization. **Anat Itzhak:** Software, Resources, Conceptualization. **Sofiya Kolusheva:** Resources, Formal analysis. **Peter Beker:** Writing – review & editing, Supervision, Resources, Project administration, Methodology, Investigation, Formal analysis.

## Declaration of competing interest

The authors declare that they have no known competing financial interests or personal relationships that could have appeared to influence the work reported in this paper.

The author is an Editorial Board Member/Editor-in-Chief/Associate Editor/Guest Editor for *Heliyon* and was not involved in the editorial review or the decision to publish this article.
